# Image Reconstruction Using Supervised Learning in Wearable Electrical Impedance Tomography of the Thorax

**DOI:** 10.3390/s23187774

**Published:** 2023-09-09

**Authors:** Mikhail Ivanenko, Waldemar T. Smolik, Damian Wanta, Mateusz Midura, Przemysław Wróblewski, Xiaohan Hou, Xiaoheng Yan

**Affiliations:** 1 Faculty of Electronics and Information Technology, Warsaw University of Technology, Nowowiejska 15/19, 00-665 Warsaw, Poland; mikhail.ivanenko.stud@pw.edu.pl (M.I.); damian.wanta@pw.edu.pl (D.W.); mateusz.midura.dokt@pw.edu.pl (M.M.); przemyslaw.wroblewski@pw.edu.pl (P.W.); 2 Faculty of Electrical and Control Engineering, Liaoning Technical University, No. 188 Longwan Street, Huludao 125105, China; hxh2022hxh@126.com (X.H.); xiaohengyan@163.com (X.Y.)

**Keywords:** capacitively coupled electrical impedance tomography, image reconstruction, inverse problem, deep neural networks, deep learning, fully connected neural networks, cGAN, medical imaging, lung imaging, pneumothorax, pleural effusion

## Abstract

Electrical impedance tomography (EIT) is a non-invasive technique for visualizing the internal structure of a human body. Capacitively coupled electrical impedance tomography (CCEIT) is a new contactless EIT technique that can potentially be used as a wearable device. Recent studies have shown that a machine learning-based approach is very promising for EIT image reconstruction. Most of the studies concern models containing up to 22 electrodes and focus on using different artificial neural network models, from simple shallow networks to complex convolutional networks. However, the use of convolutional networks in image reconstruction with a higher number of electrodes requires further investigation. In this work, two different architectures of artificial networks were used for CCEIT image reconstruction: a fully connected deep neural network and a conditional generative adversarial network (cGAN). The training dataset was generated by the numerical simulation of a thorax phantom with healthy and illness-affected lungs. Three kinds of illnesses, pneumothorax, pleural effusion, and hydropneumothorax, were modeled using the electrical properties of the tissues. The thorax phantom included the heart, aorta, spine, and lungs. The sensor with 32 area electrodes was used in the numerical model. The ECTsim custom-designed toolbox for Matlab was used to solve the forward problem and measurement simulation. Two artificial neural networks were trained with supervision for image reconstruction. Reconstruction quality was compared between those networks and one-step algebraic reconstruction methods such as linear back projection and pseudoinverse with Tikhonov regularization. This evaluation was based on pixel-to-pixel metrics such as root-mean-square error, structural similarity index, 2D correlation coefficient, and peak signal-to-noise ratio. Additionally, the diagnostic value measured by the ROC AUC metric was used to assess the image quality. The results showed that obtaining information about regional lung function (regions affected by pneumothorax or pleural effusion) is possible using image reconstruction based on supervised learning and deep neural networks in EIT. The results obtained using cGAN are strongly better than those obtained using a fully connected network, especially in the case of noisy measurement data. However, diagnostic value estimation showed that even algebraic methods allow us to obtain satisfactory results.

## 1. Introduction

Electrical impedance tomography (EIT) is a low-cost and non-invasive technique for visualizing the internal structure of the human body [1,2,3]. The image of conductance distribution is reconstructed from measurements made using the electrodes surrounding the body. The reconstruction of electric permittivity is possible when both impedance components are measured [4]. EIT relies on the different electrical properties of tissues and organs, and those properties could vary depending on tissue condition [5,6]. Different electric properties in different image regions represent the various tissues and organs. Healthy and disease-affected tissues can be localized on the image.

Image quality in EIT is limited due to very poor spatial image resolution and low sensitivity in the center of the field of view. Despite this, EIT has shown its potential in medical diagnostics [7], especially in lung ventilation monitoring, which is possible due to the high impedance of lung tissue in comparison to other tissues and to a significant difference in impedance between inspiration and expiration phases [8]. A few commercial EIT devices are available on the market currently, and some are in development [9]. In addition to lung ventilation evaluation, promising studies are devoted to lung perfusion evaluation [10]. However, other emerging medical applications of EIT, still in the research phase, are brain imaging [11], cardiac volume monitoring [12], and gastric emptying [13].

A barrier to the development of EIT is the problem of measuring very small signals. The very low signal-to-noise ratio makes it impossible to increase the number of electrodes. It leads to poor sampling of the signal at the edge of the field of view, which translates into a small number of measurements and poor spatial resolution of the image. In addition, a large difference in the measurement values for adjacent and opposite electrodes leads to poor numerical conditioning of the linear system matrix.

The new approaches for measurement in EIT were presented in the literature, for example, dual frequency EIT, multiple frequency EIT [14], and a new emerging technique named capacitively coupled electrical impedance tomography (CCEIT). The introduction of CCEIT allows for overcoming the most challenging issue of conventional EIT, such as variable electrode–skin impedance, which negatively affects measurement accuracy. Unlike EIT, where the measurement is four-electrode, CCEIT is a two-electrode measurement similar to electrical capacitance tomography (ECT). Capacitance measurement eliminates the negative effects of contact resistance as it does not require direct contact between electrodes and conductive tissue. This measurement method is less burdensome for the patient because a tomographic sensor may be constructed in the form of a wearable belt with electrodes. The tomographic sensor can also be embedded in clothes and used as a wearable tomographic sensor [15,16].

A big challenge in the EIT is to solve the inverse problem, which consists of determining the cross-sections of the tested object on the basis of measurements made using electrodes placed around this object. Ongoing research is conducted in various directions. In the analytical approach to the inverse problem, the D-Bar method, a generalization of the Calderon method, is of great interest [17,18]. The D-Bar method solves a non-linear problem, but like other algorithms, it requires regularization to reduce artifacts.

In the algebraic approach to tomographic reconstruction in EIT and CCEIT, the inverse problem is non-linear and severely ill-posed because the number of measurements is low in relation to the reconstructed image’s number of pixels. The extremely poor numerical conditioning of the sensitivity matrix makes the inverse problem difficult to regularize [19,20]. Unfortunately, the known methods of automatic selection of the regularization parameter, the cross-validation method (CGV) or the L-curve method, do not work well in the case of such poor numerical conditioning as in electrical tomography [21]. The regularization problem concerns one-step methods and iterative methods such as the Landweber algorithm.

The best image reconstruction results are obtained by non-linear algorithms, such as the Levenberg–Marquardt (LM) algorithm, in which the non-linear function is minimized [22]. Non-linear algorithms are very computationally expensive because, in each step, a sensitivity matrix (the Jacobi matrix) is determined by numerically solving the differential equation for the electric field [23]. Usage of algebraic reconstruction methods leads to a quality vs. speed trade-off: fast methods, such as linear back projection [24] or pseudoinverse, provide poor quality, and methods giving the best possible quality, such as the Levenberg–Marquardt [25] algorithm, are extremely slow to be used for real-time reconstruction.

While the progress of classical reconstruction methods is slow, deep learning methods are developing dynamically. Over the past few years, machine learning [26] and deep learning (DL), in particular, have become a new trend in tomographic image reconstruction [27]. A general description of the inverse problem in tomographic imaging and an overview of various approaches to using neural networks to solve it can be found in [28]. The idea of image reconstruction using an artificial neural network (ANN) in EIT has been known for decades [29]. With the progressive increase in the computing power of computers, interest in deep networks has increased [30]. The ability of deep neural networks to map complex non-linear functions predestines them for image reconstruction in electrical tomography. A comprehensive overview of deep learning applications in the EIT can be found in [31].

There are three main machine learning approaches to solving inverse EIT problems: single network direct reconstruction, joint reconstruction, and hybrid reconstruction [32]. The single network approach is based on using either linear or convolutional ANN. In this case, the network is trained directly on samples containing generated permittivity distributions and corresponding calculated measurements. Joint reconstruction consists of two steps: preliminary reconstruction via solving the linear approximation of the inverse problem using the traditional numerical method such as Gauss–Newton, and then enhancing the result by applying an ANN to post-produce the reconstructed image. This approach provides better stability and accuracy. An example of the hybrid approach is reconstruction, which relies on the combined usage of fully connected ANN and U-Net-based ANN. A fully connected ANN is used to obtain the preliminary physical parameter distribution [33]. Then, U-Net-based ANN is used to enhance the result of the initial reconstruction and obtain the final image.

Training ANN to reconstruct images in EIT requires a large dataset containing a correspondence between capacitance measurements and the corresponding electrical parameters’ spatial distribution. Gathering a set of patient medical records of this size is problematic. The numerical modeling of the chest phantom and the generation of measurements by solving a simple problem allow us to obtain a relatively large dataset in a reasonable time. During ANN training, it is important to artificially introduce noise into simulated data as it increases model stability and decreases the possibility of overfitting [34]. Overfitting is a well-known issue that causes the loss of ANN’s generalization ability. Additionally, it is possible to prevent overfitting by increasing the diversity of the training dataset. The ANN-based approach requires a significant amount of computational resources (memory and time) only during the training phase. The response of trained networks is very fast compared to iterative algorithms.

Examples of the ANN’s use for electrical tomography image reconstruction can be found in many scientific papers, for example, in [35,36]. Different architectures of ANNs were proposed for electrical tomography. These include feed-forward neural networks (FFNN) used in ECT [37], single-hidden layer feed-forward neural networks (SLFNs) used in EIT [35], Hopfield networks used in ECT [38], fully connected layers used in EIT [39,40], U-Net used both in ECT and EIT [33,41], a generative adversarial network used in both ECT [42] and EIT [43], and SegNet used in electrical resistance tomography (ERT) [44]. For capacitively coupled electrical resistance tomography, an approach based on convolutional neural networks (CNN) was proposed [45]. In [46], results obtained by the ANN are compared with well-validated algebraic solvers like the LM algorithm. Encoder–decoder-based networks are widely used to solve the inverse problem [47], and the brightest example of such a network is the U-Net architecture. It can be used as a sole network with slight modifications [48,49,50] and also as a part of the GAN training approach, for example, in SAR-CGAN [51]. Besides the U-Net-based approach, more complex encoder–decoder convolutional networks are used [52], which can also be used with non-uniform mesh [53].

From the point of view of the clinical application of electrical impedance tomography, there is an interest in anomaly detection in the lung region [54]. In the case of lung diagnostics, diseases such as pneumothorax and pleural effusion are particularly relevant. Recent studies have shown that these diseases cause observable changes in the measurement signal [55,56,57,58]. A large tissue conductivity difference from the normal state causes a significant change in the measured signal, negative in the case of pneumothorax and positive in the case of pleural effusion. The wearable sensor could be used by bedridden patients to monitor their lungs continuously.

### The Aim of the Work

Considering the promising properties of CCEIT, the mentioned potential of neural networks in solving the inverse problem, and the need to identify lung regions with pathological changes, we attempted to use deep neural networks to reconstruct thorax images, based on which it would be possible to identify areas affected by pathological changes. In this paper, we present a new enhanced chest phantom that takes into account the appearance of three pleural diseases (pneumothorax, pleural effusion, and hydropneumothorax) causing pathological accumulation of air or free fluid in the pleural cavity and leading to the collapse of the adjacent lung regions.

Discovering the lung lobes with disease presence is described in [59]. However, the presented method allows obtaining only five average conductivity values in such regions, and it is based on the strong assumption that the geometry of lobes is known.

We propose a modification of the thorax numerical phantom presented in [60], in which the lung displacement in the pleural cavity and shape deformation are modeled using elliptical regions. We assume individual differences in the shape and position of the organs in the patient’s chest. The use of simple geometric elements in chest modeling arises from the need to build an appropriate physical phantom to carry out real measurements. The proposed numerical phantom is suitable for reuse by other researchers.

In our work, the image quality of a 32-electrode CCEIT was studied, whereas in other papers, a smaller number of electrodes is used. For example, in the study [58] devoted to pleural disease discovery, the 22-electrode sensor was modeled. We want to show that the use of ANNs in the reconstruction of electrical tomography images using the sensor with 32 area electrodes allows us to obtain more detailed images with 64 × 64 resolution.

The use of ANN in electrical tomography image reconstruction is commonly known. However, in this paper, we use ANN-based reconstruction for the first time in the case of a lung model with regions affected by pleural diseases. This work is focused on exploring the required ANN complexity depending on the assumed level of noise. To achieve that goal, we have chosen a very simple shallow and sophisticated deep convolutional neural network, a fully connected network (FCNN), and a conditional generative adversarial network (cGAN), respectively. Pixel-based metrics are used to assess the results in most papers. In this work, we have proposed the use of the diagnostic value measured using the area under the receiver operating characteristic (ROC) curve (AUC) metric to evaluate image reconstruction quality.

## 2. Machine Learning Approach in CCEIT

This work uses supervised learning, which requires a training dataset containing ordered input and output data pairs. In electrical tomography, a pair of data is an image of a tomographic cross-section of the tested object and electrode measurements for this object.

In CCEIT, the input data are the measurements of inter-electrode capacitances, and the output data are the images of the permittivity distribution in the examined space. In the case of patients’ thorax exams, it is unrealistic to collect a large amount of training data in the early stages of the study. Due to the large size of the training set, we decided to generate data using numerical simulation.

The ECTsim software package, dedicated to the Matlab environment, was used to generate training data [61]. The ECTsim package enables numerical modeling of electrical capacitance tomography in 2D and 3D geometry, but it can also be used to simulate capacitively coupled electrical impedance tomography [12,13]. In the ECTsim package, calculations are performed in the complex domain. The medium is described by a complex permittivity:

(1) $$\varepsilon =\varepsilon^{\prime }-j\frac{\sigma }{\omega }$$

where $\varepsilon^{\prime }$ is permittivity, σ is conductivity, and ω is the angular frequency. The measurements are calculated using the finite volume method [62] from the Gauss law equation:

(2) $$C_{m\left(i,j\right)}=\frac{1}{V_{i}-V_{j,\;j\neq i}}\overset{}{\underset{\partial \Omega_{j}}{∯}}\varepsilon \left(r\right)E\left(r\right)ds$$

where ε(r) is complex permittivity at position r in the examined volume, E(r) is an electric field, $\partial \Omega_{j}$ is a surface surrounding the measuring electrode, ds is a normal vector to a small element of a surface, $V_{i}$ is a potential at the i-th electrode, and $C_{m\left(i,j\right)}$ is a complex capacitance measured for a pair of electrodes (i,j). The ECTsim toolbox allows defining a model describing the permittivity and conductivity of the object and uses a finite volumes method to calculate corresponding mutual capacitances between all defined electrodes. The simulation of the electric field is performed on a non-uniformly refined Cartesian mesh. Solving the forward problem with ECTsim requires defining the geometrical and electrical parameters of the model, which should contain electrodes, isolation, and objects in the field of view.

### 2.1. Model of the Human Thorax

**Figure 1:**
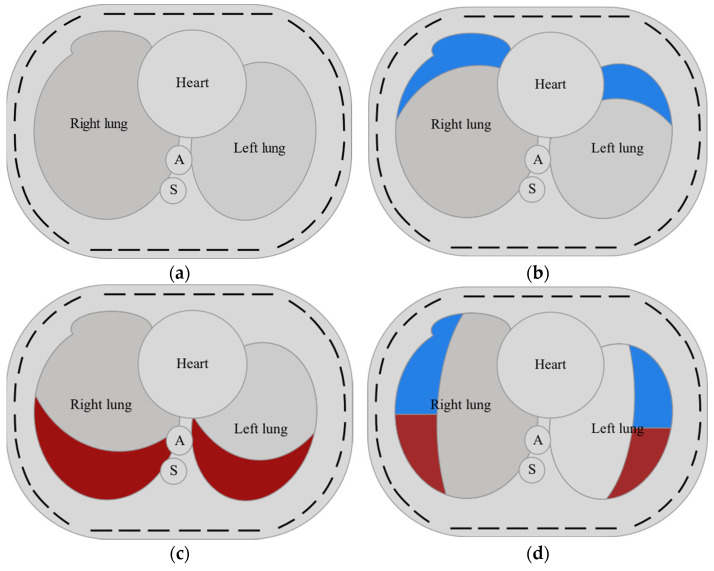
Model of a transversal slice of a human thorax with lungs, heart, aorta (A), and spine (S) (**a**). Model of both lungs regionally affected by pneumothorax (**b**), pleural effusion (**c**), and hydropneumothorax (**d**). Pneumothorax and pleural effusion regions are shown, respectively, in blue and red.

A model of a 2D slice of the human thorax, proposed in [60], was used in the generation of the training dataset. The proposed thorax phantom is based on axial lung CT slice segmentation [63] and presents the patient lying on his back. The model includes the heart, aorta, spine, and lungs (Figure 1a). A sensor with 32 electrodes was used. Permittivity and conductivity values of the model elements are given in Table 1 and Table 2. Lung tissue permittivity and conductivity values (Table 1) for inspiration and expiration phases are defined for excitation frequency 100 MHz for healthy lungs and two conditions: pneumothorax (further referred to as illness A) and pleural effusion (illness B) [56]. The lung and pleura can be affected by either one or both conditions. Pneumothorax occurs when there is a pathological accumulation of air between the collapsed lung tissue and the interior wall of the thorax. When a patient is lying in a supine position, the air in the pleural cavity moves towards the anterior chest wall, which corresponds to the top of the image. Therefore, pneumothorax is modeled by generating an ellipse shifted to the bottom and center of the model. The intersection of this ellipse with the lung represents the partially collapsed lung tissue, and the rest of the thorax represents the air trapped in the pleural cavity. Pleural effusion is defined as the pathological accumulation of excessive fluid in the pleura. In the supine position, free fluid tends to collect in the dorsal parts of the pleura, which corresponds to the bottom of the image. Pleural effusion is modeled in a similar way as pneumothorax, but the ellipse is shifted to the top and center of the image. Hydropneumothorax occurs when the pleura is affected simultaneously by both illnesses (the concurrent presence of air and fluid in the pleural cavity). In this case, the pleural effusion is almost perfectly horizontal in the supine position, with no characteristic meniscus. Therefore, the healthy part of the thorax is represented by the intersection of the lung with an ellipse shifted towards the center of the model. The pleura is divided by a straight line into the upper part corresponding to the pneumothorax and the lower part corresponding to the pleural effusion. The dielectric properties of the effusion fluid are close enough to those of blood with extremely low red blood cell amounts. According to cow blood-based studies, blood’s electrical parameters (relative permittivity and conductivity) depend on the hematocrit level [64]. In accordance with human blood-based studies, we can take the conductivity value for hematocrit 0 to be equal to 1.4 S/m [65]. This value is consistent with the value obtained from cow blood, so for the purpose of this work, we can assume a permittivity value of 70. Both conditions imply a reduction in the image region with electrical properties corresponding to the healthy lung and growth in the area with abnormal electrical properties.

When generating the training set, possible individual changes were taken into account, i.e., the location and size of the lungs, the location and size of the heart, aorta, and spine. A certain range of change in the center position and the angle of rotation was adopted for all organs. All organs could also change their size, but the change in both axes was independent—all organs were modeled using ellipses. A uniform probability distribution was used for all ranges when selecting parameters from the range. Whether the lungs were healthy or affected by diseases A or B was randomized. The size and location of the illness-affected regions were randomized, with pneumothorax appearing only at the top (front of the supine patient) and effusion at the bottom of the image (back of the supine patient).

In ECTsim, the numerical simulation is performed on a dense, non-uniform mesh, but the reconstruction is conducted on a reduced mesh. In this work, the matrix of the reconstructed image was 64 × 64 pixels. Since only pixels in the field of view of the tomographic probe are reconstructed, the number of pixels that are reconstructed is smaller and equal to 1856 for the adopted model. This significantly reduces the size of the output data vector of the neural network.

### 2.2. Measurement Simulation in Electrical Capacitively Coupled Electrical Impedance Tomography

By solving a forward problem in CCEIT, i.e., solving the equation for the electric field, it is possible to calculate capacitance measurements between electrodes based on the known distribution of complex electrical permittivity in the examined volume. The forward problem can be written as:

(3) c=f(ε)

where f is a non-linear vector function, $c\;\in R^{M}$ is a complex capacitance vector, and $\varepsilon \;\in \;R^{N}$ is a complex electrical permittivity vector. It is possible to use the linear approximation of the non-linear function f as follows:

(4) $$c_{M\times 1}=S_{M\times N}\varepsilon_{N\times 1}$$

where S is the so-called sensitivity matrix, which is a Jacobian of the capacitance with respect to pixel values, representing electrical permittivity [66]. By having K electrodes and measuring the capacitance between each pair of electrodes, it is possible to acquire:

(5) M=K(K−1)/2 

measurements [67]. If the measurement for a pair of electrodes is made twice by exchanging the roles of the electrodes (the exciting electrode changes with the measuring electrode), there are twice as many measurements. For K=32 electrodes, there are M=992 capacitance measurements with repetitions. The size of the measurement vector corresponds to the size of the input of the neural network. The number of capacitance measurements and the number of pixels to reconstruct determine the size of the model. In the experiments conducted, the size of the input vector was M = 992, and the size of the output vector was N = 1856.

### 2.3. Training Dataset

The decision on the number of samples in the training dataset is one of the most difficult, as there is no accurate method to estimate the necessary dataset size. However, our experiments show that a model with an input size of 992 and an output size of 1856 trained on a dataset containing around 200,000 samples provides good results. The dataset should include randomly generated samples representing healthy lungs and lungs partially affected by the illnesses. Because lungs can be affected by one or both conditions, it is possible to have 16 different cases depending on which lung is affected by which condition. We created a dataset that evenly represented all 16 cases. Additionally, to mitigate overfitting, we introduced samples with random ellipses.

For ANN training, it is necessary to divide the whole dataset into training, validation, and testing subsets. The training subset is used as the source of experience during model training. The validation subset is used to monitor the main metric while training, and the testing subset is used for the final model performance evaluation. Two datasets were generated with the model shown in Figure 1. Subsequently, samples with random ellipses were introduced into datasets, resulting in:

- The training set contained 9375 samples from each class and 44,000 samples with random ellipses, for a total of 194,000 samples. During training, this set was randomly split on the fly into learning and validation subsets with a ratio of 75:25.
- The test set contained 2500 samples from each class and 2500 samples with random ellipses, for a total of 42,500 samples.

**Figure 2:**
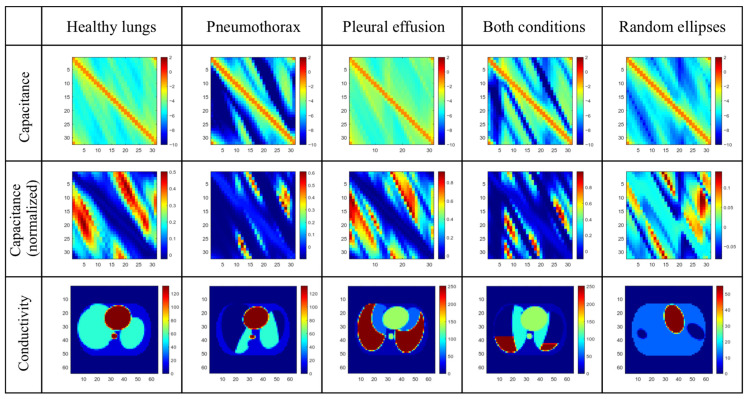
Dataset samples: capacitance measurements (log scale), normalized capacitance measurements (linear scale), and corresponding conductivity distribution.

Each sample of the dataset generated by ECTsim contains a complex capacitance measurements vector of size 992 and a complex electrical permittivity values vector for 1856 pixels from the field of view. Due to the limitations of the PyTorch framework, which is capable only of ANN training on real numbers, only the imaginary components of capacitance measurements and electrical permittivity were taken for ANN training purposes. Capacitance measurements depend on the electrode’s relative position and the electrical properties of the material inside the sensor. To mitigate mutual electrode arrangement influence, one can normalize measurements by calculating capacitance for the empty sensor and the sensor filled with a high-conductivity material. Capacitance measurements can be stored as a 2D matrix where the column and row indices correspond, respectively, to the excitation and sensing electrodes. Example color maps of conductivity distributions and resulting capacitance measurements (imaginary component) are presented in Figure 2.

### 2.4. ANN Architecture Used

Two different neural network architectures were used in the experiments: a fully connected neural network (FCNN) similar to EIT-4LDNN [39] and a convolutional network based on a conditional generative adversarial network (cGAN) architecture. We will show that with the usage of modern ANN training techniques, such as the AdAM optimization algorithm [68] and batch normalization layers [69], it is possible to obtain satisfactory results with the usage of a simple, fully connected ANN with one hidden layer.

**Figure 3:**
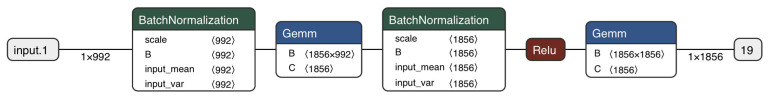
FCNN architecture: Gemm—linear layers; Relu—ReLU activation.

As a fully connected NN (FCNN), we used the network architecture shown in Figure 3. It takes a vector of size 992 as an input, then applies batch normalization to the input, applies one linear layer (implemented as a general matrix multiply (GeMM) algorithm) with batch normalization and rectified linear unit (ReLU) activation, and then one more linear layer.

**Figure 4:**
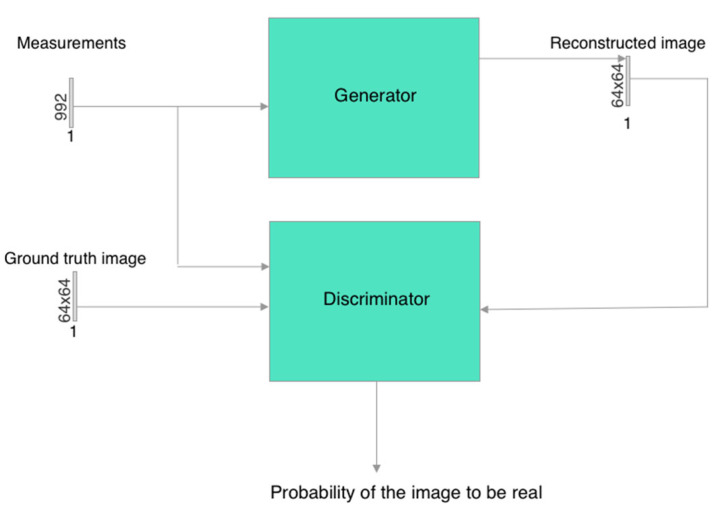
cGAN training scheme.

**Figure 5:**
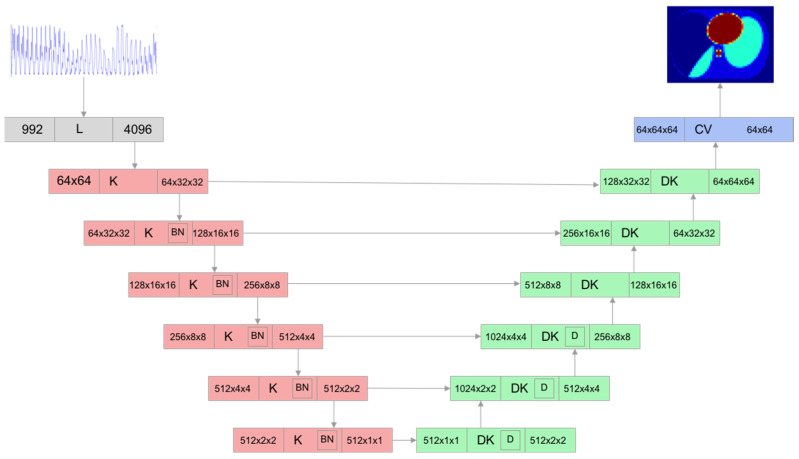
cGAN generator architecture: K—convolutional block, DK—deconvolutional block, CV—convolutional layer, L—linear layer.

**Figure 6:**
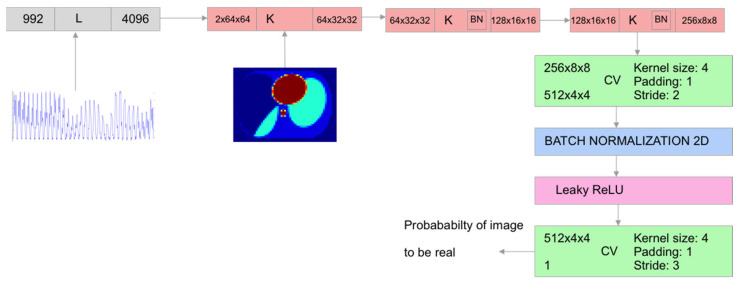
cGAN discriminator architecture: K—convolutional block, CV—convolutional layer, L—linear layer.

**Figure 7:**
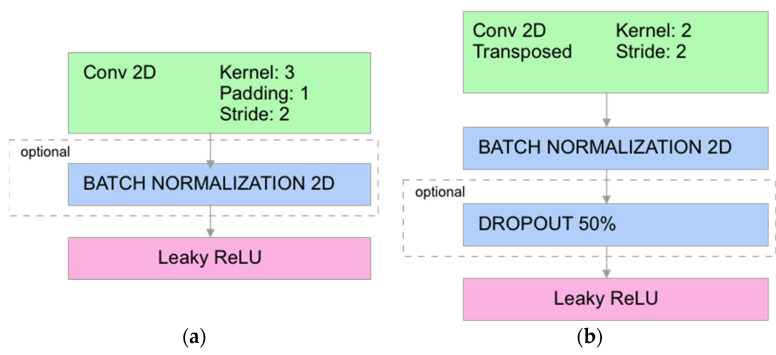
cGAN generator blocks: (**a**) convolutional (K) and (**b**) deconvolutional (DK).

A different model used in this work was a convolutional network based on the cGAN architecture (Figure 4). It consisted of a U-Net-based generator (Figure 5) and a simple discriminator consisting of several convolutional layers (Figure 6). The convolutional and deconvolutional blocks were used as the building blocks for the generator and discriminator, as shown in Figure 7. Usually, the generator in the cGAN approach takes a random latent vector and condition vector as input and generates an image as an output. The latent vector is intended to provide sufficient intraclass variability, which helps to obtain a more reliable output. Such an approach works well in the case of a relatively small condition vector, for example, as in the case of 12 electrodes corresponding to the size 66 of the condition vector [70]. When the condition size increases, the network starts to lose stability, and some modifications are required. Our experiments found that using the Pix2Pix approach [71] allowed us to create a stable cGAN network in the case of 992 measurements. Pix2Pix approach implies two main modifications to the traditional cGAN model: usage of dropouts inside the generator instead of latent vectors and modification of the generator loss function, which is based on adding L2 and L1 loss components referencing true images for the given condition as follows:

(6) $$f\left(l_{r},l_{p},y_{r},y_{p}\right)=\mathrm{BCR}\left(l_{p},l_{r}\right)+100\mathrm{MSE}\left(y_{r},y_{p}\right)+100\left|y_{r}-y_{p}\right|\;$$

where $l_{r}$ is the reference value of the probability that the image is true, and $l_{p}$ is the probability value of the image being true, determined by the discriminator. $y_{r}$ and $y_{p}$ are reference and predicted images, respectively; BCR is a binary cross-entropy and MSE is a mean square error.

We divided the whole dataset into training and testing subsets to train the reconstruction model as described above. A binary cross-entropy was used as a loss function while training the discriminator, and the AdAM was used as an optimizer.

### 2.5. Reconstruction Quality Assessment

To correctly assess ANN training quality, it is necessary to divide the dataset into three parts: training, validation, and test subsets. The training subset is used for the actual ANN training. The validation subset allows adopted metrics to be calculated on the fly during training, and the test dataset is necessary for the final evaluation of the training result.

A widespread quality evaluation strategy in EIT image reconstruction mainly focuses on adopting simple pixel-to-pixel metrics such as average relative error, L2 norm (RMSE), or 2D correlation coefficient [39,70]. Such metrics allow estimating conditional consistency—the likelihood of reconstructing the same image for the same given input [72], but they are limited because averaging the metric on a test dataset does not take into account outlying cases. Therefore, in this paper, we propose calculating metric dispersion in addition to the metric mean value on the test dataset.

In this work, we use the following pixel-based metrics to evaluate reconstruction quality: root mean square error (RMSE), 2D correlation coefficient (CC), peak signal-to-noise ratio (PSNR), and structural similarity index measure (SSIM). These metrics are defined as follows:

(7) $$\mathrm{RMSE}\left(\hat{y},y\right)=\sqrt{\frac{1}{N}\overset{N}{\underset{i=1}{\sum }}{\left|y_{i}-{\hat{y}}_{i}\right|}^{2}}\;$$

(8) $$\mathrm{CC}\left(\hat{y},y\right)=\frac{\sum_{i=1}^{N}\left({\hat{y}}_{i}-\bar{\hat{y}}\right)\left(y_{i}-\bar{y}\right)}{\sqrt{\sum_{i=1}^{N}{\left({\hat{y}}_{i}-\bar{\hat{y}}\right)}^{2}\sum_{i=1}^{N}{\left(y_{i}-\bar{y}\right)}^{2}}}$$

(9) $$\mathrm{PSNR}\left(\hat{y},y\right)=10\log_{10}N\frac{\max\hat{y}}{\sum_{i=1}^{N}{\left|y_{i}-{\hat{y}}_{i}\right|}^{2}}$$

(10) $$\mathrm{SSIM}\left(\hat{y},y\right)=\frac{\left(2\mu_{y}\mu_{\hat{y}}+c_{1}\right)\left(2\sigma_{y\hat{y}}+c_{2}\right)}{\left(\mu_{y}+\mu_{\hat{y}}+c_{1}\right)\left(\sigma_{y}^{2}+\sigma_{\hat{y}}^{2}+c_{2}\right)}\;\;$$

where

N—number of pixels,

$y_{i}$—expected conductivity value at pixel i,

${\hat{y}}_{i}$—reconstructed conductivity value at pixel i,

$\mu_{y}$—mean value of vector y,

$\mu_{\hat{y}}$—mean value of vector $\hat{y}$,

$\sigma_{y}^{2}$—variance of vector y,

$\sigma_{\hat{y}}^{2}$—variance of vector $\hat{y}$,

$\sigma_{y\hat{y}}$—covariance of vectors y and $\hat{y}$,

$c_{1}$ and $c_{2}$—stabilization variables to prevent division by zero on weak denominators.

### 2.6. Image Diagnostical Value Evaluation Using ANN Classifier

Considering the medical applications of EIT, it is implied to evaluate its model diagnostic value. For this purpose, in this work, we proposed the following procedure: Labeled samples corresponding to different medical conditions are entered into the training dataset. The classifying neural network is trained on the generated image data (true images). The classifying neural network is verified on reconstructed images. Comparison of the classification results allows us to assess the diagnostic value of the reconstructed images.

A well-known approach to the comparison of classification models is the use of receiver operating characteristic curves (ROC). The ROC curve demonstrates the diagnostic ability of a binary classifier depending on changes in the discrimination threshold. The value of the AUC (area under the ROC curve) metric shows the model’s performance and ranges from 0 to 1, where 1 means perfect performance, 0.9 means very good performance, and 0.5 means no performance.

For multi-class classification, as in our case, the analysis of the AUC metric needs to be adjusted. In our model, we have 16 classes of lung status, starting with a class when both lungs are healthy and ending with a class when both lungs are affected by both diseases. One additional class (17th) corresponds to random ellipses in the thorax phantom. The OvR (one versus the rest) method was used, which consists of calculating the ROC curve for each of the 17 classes, where in each step, a given class is considered a positive class and the remaining classes are given a negative weight. The average ROC curve is used to evaluate the multi-class classifier [73].

**Figure 8:**
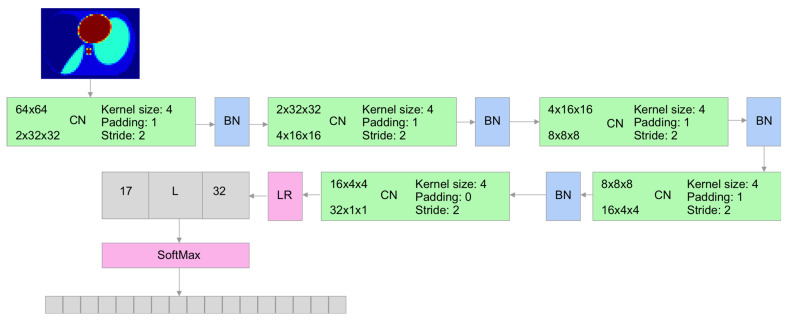
ANN classifier architecture.

To evaluate reconstruction quality, we used the classifier ANN with the architecture shown in Figure 8. A conductivity vector dataset of size 1856 reshaped to a 64×64 matrix is used as an input to the ANN classifier. The classifier produces the probability of belonging to one of the 17 classes. Because of the two-dimensional input, we selected the following network architecture: We used 2D convolutions with batch normalization and then applied a fully connected layer with softmax activation to produce a vector of probabilities. Softmax activation has the property that the sum of all vector elements is one. Therefore, it is possible to interpret those values as probabilities.

The model was trained on generated images from training and validation subsets and corresponding labels saved during dataset generation. Those samples were divided into training and validation parts in a ratio of 75:25. Binary cross-entropy was used as a loss function, and the AdAM optimizer was used as an optimizer.

## 3. Results

**Figure 9:**
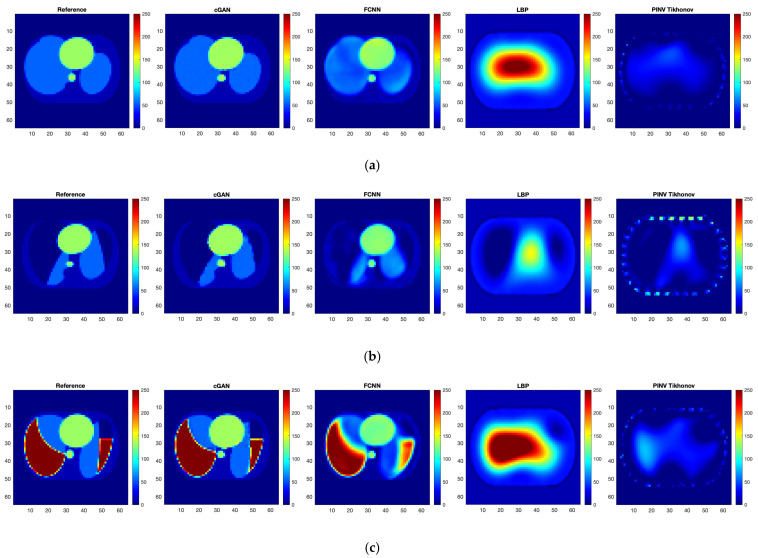

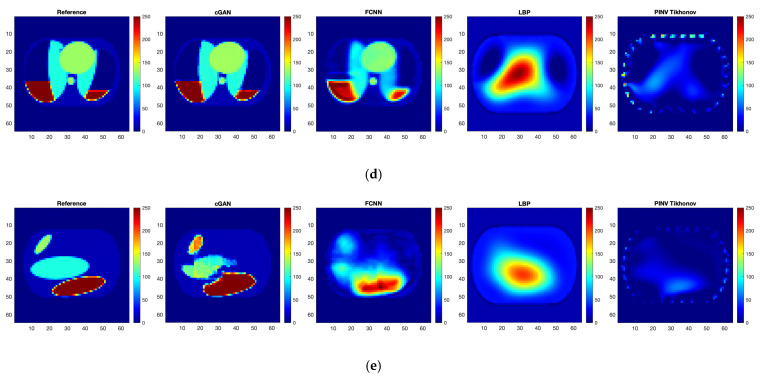
Conductivity in a thorax slice: (**a**) healthy lungs; (**b**) both lungs affected by pneumothorax; (**c**) both lungs affected by pleural effusion; (**d**) lungs affected by hydropneumothorax; (**e**) random ellipses in the thorax. From left to right: ground truth image; image reconstructed by FCNN, cGAN, LBP, and TPINV.

After training the FCNN and cGAN networks, reconstruction quality evaluations have been carried out. We reconstructed images from the testing dataset containing 42,500 samples using trained ANNs and selected algebraic methods (Figure 9). Because the response of ANN is very fast, we compared ANN reconstruction with fast one-step algebraic methods:

- The linear back projection (LBP) given by
  (11) $$\varepsilon =\varepsilon_{min}+{\tilde{S}}^{T}c_{n},$$
  where ${\tilde{S}}^{T}$ is a transpose of the normalized sensitivity matrix and $c_{n}$ is a vector of normalized measurements.
- The pseudoinverse with Tikhonov regularization (TPINV) given by
  (12) $$\varepsilon ={\left(S^{T}S+\alpha I\right)}^{-1}S^{T}c_{n}.$$

The regularization parameter α was chosen heuristically as 10^−9^ to achieve the best possible results.

**Figure 10:**
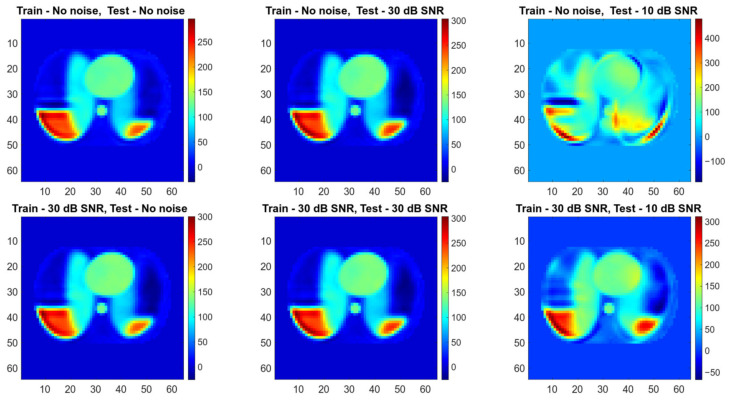
Conductivity in a thorax slice reconstructed by FCNN for different level of noise added to measurements in training and testing datasets.

**Figure 11:**
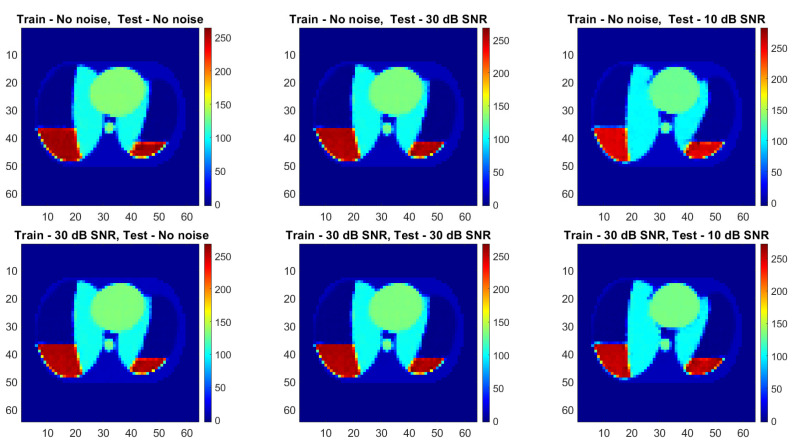
Conductivity in a thorax slice reconstructed by cGAN for different level of noise added to measurements in training and testing datasets.

**Figure 12:**
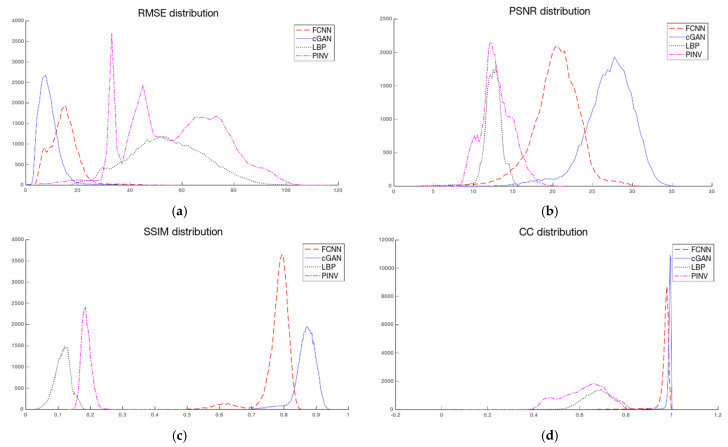
Distribution of the image quality norm for the elements of the testing dataset: (**a**) RMSE, (**b**) PSNR, (**c**) SSIM, and (**d**) 2D correlation. Image reconstruction methods: FCNN (red dashed line), cGAN (blue solid line), LBP (black dotted line), pseudoinverse with Tikhonov regularization (TPINV) (magenta dashed-dotted line).

The abovementioned image quality measures, such as RMSE, PSNR, SSIM, and 2D correlation coefficient, were computed for each pair of ground truth and reconstructed images. The mean value, standard deviation, and median over the testing dataset were calculated (Table 3). Image quality norms for the testing data with a high level of noise (30 dB) calculated for an image reconstructed using ANNs are given in Table 4. It is easy to see that reconstruction with the use of ANNs gives significantly better results than algebraic methods. The image quality norms for reconstruction using very noisy measurement data (30 dB and 10 dB) obtained by neural networks are better than the norms obtained for low-noise data by classical methods (LBP, TPINV). And among ANNs, the cGAN-based network produces more accurate images. It is easy to see that a cGAN-based network allows us to obtain very good results even in the case of very high noise (10 dB). Introducing noise during training makes results from both FCNN and cGAN-based networks even better and allows us to obtain images comparable to those obtained from measurements without noise. However, the simple network (Figure 10) cannot achieve the level of quality produced by the complex convolutional network (Figure 11). The histograms of the distribution of image distance measures are shown in Figure 12.

**Figure 13:**
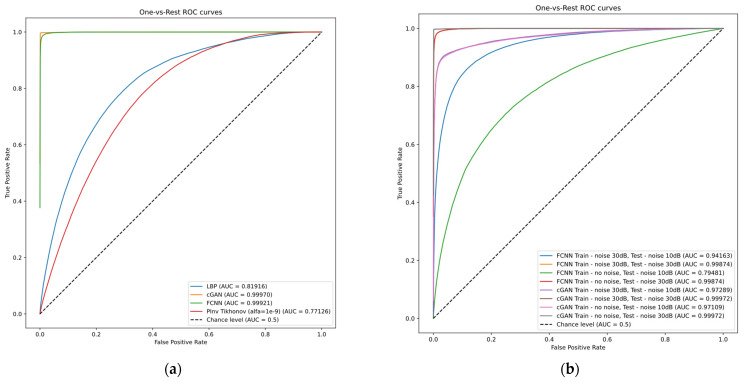
“One-versus-rest” integral ROC curves representing the classifier performance: (**a**) training and testing without noise, (**b**) training and testing with noise introduction.

The diagnostic value of reconstructed images was evaluated using the classifier network trained on ground truth images. The OvR (one versus the rest) method was used to assess multi-class classification. We calculated mean ROC curves for each reconstruction method without introducing noise into measurements and then calculated corresponding AUC metric values (Figure 13a). Cases involving the introduction of noise into training and testing datasets were also evaluated (Figure 13b). The resulting values were added to Table 3 and Table 4, respectively. The obtained values show that ANN-based image reconstruction methods allow for a comparably high ability of the classifier to recognize lung illness conditions. However, it is interesting that even one-step algebraic methods allow for obtaining results confirming that the reconstructed images contain information about the presence of the disease in lung regions. Differences in the conductivity values in the reconstructed images resulting from the presence of the disease are sufficient to identify the disease entity.

## 4. Discussion

In our work, we have shown the reconstruction of human thorax numerical phantom images in 32-electrode CCEIT. The two artificial neural networks were used to solve an inverse problem. As expected, the more complex NN based on the CGAN architecture achieved better results than the simple, fully connected NN (FCNN). The FCNN produces acceptable results but has reduced generalization ability. This is evidenced by the second peak, for a lower SSIM value, visible in the SSIM measure histogram for the test dataset (Figure 12c). This peak corresponds to the images of random ellipses. The CGAN-based network does not show any irregularities on metrics histograms.

Diagnostic ability evaluation showed very good classification performance on ANN-reconstructed images. However, unexpectedly, the reconstruction with the use of simple one-step algebraic methods also showed acceptable performance. That leads to the hypothesis that it is potentially possible to detect illnesses based on simple reconstruction. Also, it may be possible to create a classifier network able to detect illness conditions directly from measurements.

Algebraic methods require both real and imaginary parts of measurements represented by complex numbers. However, ANN training on complex numbers is only at its beginning. Therefore, we trained networks only on the imaginary part of the measurements and electrical permittivity. It might be interesting to train a network using complex input and output and compare the robustness of such a complex weighted ANN.

## 5. Conclusions

The use of CCEIT in wearable solutions requires a fast and computationally efficient reconstruction method. Our work shows that such a solution may be obtained with the use of an artificial neural network. We have developed a numerical phantom modeling the transversal slice of the human thorax, allowing simulation of different illness conditions arising in the regions of the image showing a conductivity distribution in the thorax. Such conditions correspond to the cases of lung reduction on the image and the appearance of regions with extremely high or low conductivity on the image. We have shown that numerical simulation of the reconstruction process with an ANN-based approach shows very promising results: while the reconstruction speed is comparable to a one-step algebraic approach, reconstruction quality, assessed with the use of image distance measures, is much higher in the case of the ANN-based approach.

In addition to the evaluation of results based on image quality metrics, an approach based on the assessment of the classifier’s ability to recognize a disease state using reconstructed images was proposed. The assessment using the classifier showed that even the images reconstructed by the simplest reconstruction methods still contained information about the location of the zones with changed conductivity.

As a result of the analysis of neural network models, it was concluded that a more complex network based on the cGAN architecture gives better reconstruction results, although the simplest possible network gives relatively good results.

Due to the limitations of the tools used, training of deep networks was carried out using only an imaginary part of capacitance and electrical permittivity. However, the real processes occurring during electrical capacitance tomography are better described by complex numbers.

Due to the simulation nature of the above work, the obtained results cannot be directly used for medical imaging. It is, therefore, necessary to conduct further research, both in simulation and, if possible, on real data. Based on the obtained results, it can be concluded that there are three directions for further research: clarification and extension of the model based on convolutional networks, ANN training using complex numbers, and examination of the classification possibilities of data reconstructed by classical reconstruction algorithms.

## Figures and Tables

**Table 1:** Lung tissue permittivity and conductivity values at 100 MHz.

	Healthy Lungs		Pneumothorax	Pleural Effusion
	Inspiration	Expiration		
Relative permittivity	31.6	67.1	1	70
Conductivity, S/m	0.306	0.559	10^−15^	1.4

**Table 2:** Model component permittivity and conductivity values.

Component	Permittivity	Conductivity
Electrodes (metal)	1	0.0643
Isolation (plastic)	2	10^−21^
Spine	10.53	0.0643
Heart, Aorta	90.8	0.733
Fat	12.7	0.068

**Table 3:** Image quality norms (RMSE—root-mean-square error; PSNR—peak signal-to-noise ratio; SSIM—structural similarity index; CC—2D correlation coefficient; DV—diagnostic value) for the testing dataset. SNR = 60 dB. The mean value of the norm, the median, and the standard deviation for the elements of the testing dataset.

Method	RMSE			PSNR			SSIM			CC			DV
	µ	M	σ	µ	M	σ	µ	M	σ	µ	M	σ	AUC
FCNN	14.61	14.49	5.49	20.37	20.55	2.92	0.78	0.79	0.05	0.96	0.97	0.05	0.99
cGAN	8.86	8.17	4.13	27.06	27.39	3.07	0.87	0.87	0.03	0.98	0.99	0.03	0.99
LBP	54.37	53.94	14.4	12.45	12.52	1.23	0.11	0.12	0.02	0.68	0.68	0.06	0.82
TPINV	57.22	57.48	18.16	12.93	12.73	1.97	0.19	0.19	0.02	0.62	0.63	0.1	0.77

**Table 4:** Image quality norms (RMSE—root-mean-square error; PSNR—peak signal-to-noise ratio; SSIM—structural similarity index; CC—2D correlation coefficient; DV—diagnostic value) for the testing dataset with Gaussian noise added to measurements during training and testing. The mean value of the norm, the median, and the standard deviation for the elements of the testing dataset.

Method	RMSE			PSNR			SSIM			CC			DV
	µ	M	σ	µ	M	σ	µ	M	σ	µ	M	σ	AUC
Training—no noise, Testing—30 dB SNR													
FCNN	14.84	14.76	5.53	20.44	20.66	3.02	0.78	0.79	0.05	0.96	0.97	0.05	0.99
cGAN	9.45	8.96	4.37	26.9	27.17	2.96	0.87	0.87	0.03	0.98	0.99	0.03	0.99
Training—no noise, Testing—10 dB SNR													
FCNN	62.51	61.18	14.17	11.2	11.22	1.83	0.48	0.48	0.05	0.68	0.7	0.13	0.79
cGAN	18.00	17.36	7.37	21.67	22.01	3.67	0.81	0.82	0.04	0.95	0.96	0.06	0.97
Training—30 dB SNR, Testing—30 dB SNR													
FCNN	15.01	14.92	5.52	20.28	20.5	2.97	0.77	0.79	0.05	0.96	0.97	0.05	0.99
cGAN	9.58	9.07	4.43	26.8	27.06	2.98	0.87	0.87	0.03	0.98	0.99	0.03	0.99
Training—30 dB SNR, Testing—10 dB SNR													
FCNN	27.55	27.13	6.61	14.96	15.12	2.38	0.64	0.65	0.06	0.89	0.91	0.08	0.94
cGAN	17.54	16.86	7.29	22.03	22.41	3.74	0.82	0.82	0.04	0.95	0.97	0.06	0.97

## Data Availability

Not applicable.
